# Exploring the association of serum prolactin with serum glucose levels and clinical findings in a cohort of patients with early rheumatoid arthritis

**DOI:** 10.1186/s42358-024-00394-8

**Published:** 2024-08-07

**Authors:** Lidiana Bandeira de Santana, Thomas Alves de Souza Lima, Amanda Rodrigues Costa, Leticia Assad Maia Sandoval, Talita Yokoy de Souza, Licia Maria Henrique da Mota, Luciana Ansaneli Naves

**Affiliations:** 1 University Hospital of Brasilia, Brasilia, Brazil; 2 Faculty of Health Sciences, University of Brasilia, Brasilia, Brazil; 3 Legislative Consultancy in Health, Federal Senate, Brasilia, Brazil; 4 Faculty of Medicine, University of Brasilia, Brasilia, Brazil

**Keywords:** Rheumatoid arthritis, Prolactin, Disease activity, Plasma glucose, Fatigue

## Abstract

**Background:**

In the context of rheumatoid arthritis and its systemic inflammatory implications, there is an increasing interest in investigating the role of prolactin in the clinical and metabolic aspects of the disease. This study aimed to explore the potential links between serum prolactin levels, serum glucose levels, and the clinical manifestations of arthritis.

**Methods:**

This exploratory, cross-sectional, observational study focused on women diagnosed with rheumatoid arthritis. The research involved assessing prolactin and blood glucose concentrations, alongside specific clinical traits such as disease-related inflammation, morning stiffness, and fatigue intensity. The presence of changes in serum prolactin (PRL) was initially compared among the groups based on disease activity intensity. Using a multinomial regression analysis, the study analyzed the impact of predetermined clinical and metabolic factors on various categories of prolactin concentration.

**Results:**

Out of the 72 participants included in the study, hyperprolactinemia was detected in 9.1% of the sample. No differences in serum PRL were identified among the evaluated groups based on disease activity. Following multivariate analysis, no statistically significant differences were identified for the outcomes of inflammatory activity and morning stiffness within each PRL category when compared to the reference category for PRL. There was no increased likelihood of encountering blood glucose levels below 100 mg/dl among individuals with higher prolactin concentrations compared to those in the lowest prolactin category (OR 5.43, 95% CI 0.51–58.28). The presence of clinically significant fatigue revealed a higher likelihood of encountering this outcome among patients with intermediate PRL values (prolactin categories 7.76–10.35 with OR 5.18, 95% CI 1.01–26.38 and 10.36–15.29 with OR 6.25, 95% CI 1.2–32.51) when compared to the reference category.

**Conclusions:**

The study found no discernible correlation between prolactin concentrations and worse scores for inflammatory activity of the disease, nor between prolactin concentrations and serum glucose levels. The findings regarding fatigue should be approached with caution given the exploratory nature of this study.

**Supplementary Information:**

The online version contains supplementary material available at 10.1186/s42358-024-00394-8.

## Introduction

Prolactin (PRL) is a hormone primarily secreted by the anterior pituitary [1]. Its primary hormonal role is to stimulate breast development, thus ensuring galactopoiesis [2]. However, PRL has been associated with numerous other biological functions over the years, particularly in the context of metabolic and immunological activities [3]. In addition to pituitary PRL, other sources of this hormone have been identified, including cells of the immune system [4]. The PRL receptor is part of the cytokine receptor superfamily [5] and is also expressed in other regions of the nervous system, immune cells, the liver, the pancreas, and adipose tissue [6].

Dopamine, which is the primary regulator of pituitary PRL [7], may also be involved in the modulation of the immune and metabolic systems directly through its action on dopaminergic receptors [8] found in regions where dopamine synthesis occurs, such as adipose tissue, the pancreas, immune cells, and synovial fibroblasts [9]. Dopaminergic transmission generally depends on the dopamine concentration [10] and subtype of receptor involved and is further regulated by different heteromers formed by dopamine receptors and other receptors [9], sometimes promoting antagonistic dopaminergic effects. The interaction of dopamine with peripherally derived PRL still requires further investigation [11], and the actions of dopaminergic agonists and antagonists cannot be attributed solely to variations in serum PRL levels.

In the periphery, other factors can affect the autocrine and paracrine actions of PRL, such as the conversion of PRL into vasoinhibins, which are fragments with molecular weights between 11 and 18 kDa and can exert opposite effects to those of full-length PRL. In joint tissues, full-length PRL is associated with angiogenic effects, whereas vasoinhibins, on the other hand, are linked to pro-inflammatory and anti-angiogenic effects [12].

Distinct actions of PRL can be observed based on the concentration of this hormone, with high concentrations potentially eliciting an inhibitory immune response and low concentrations exhibiting an immune-stimulatory activity [13].

Among autoimmune diseases, rheumatoid arthritis (RA) stands out due to its chronic inflammatory nature, affecting joints and the systemic systems [14, 15]. Systemic inflammation in RA promotes increased insulin resistance [16], and long-term inflammation, including that in adipose tissue [17], may contribute to dysfunction of pancreatic β-cells and affect the hepatic pathway of glucose metabolism [18]. Glucose homeostasis in these patients may still be affected by the habitual use of glucocorticoids employed for disease activity control [19]. Cardiovascular risk in RA is also associated with markers of systemic inflammation [20], while the protective role of high-density lipoprotein (HDL-c) appears to be compromised by inflamed and metabolically dysfunctional adipose tissue [21].

Several small-scale clinical studies have linked serum PRL levels with RA disease activity, but results have been conflicting [12, 22]. Other studies reveal significantly higher PRL receptor expression in the synovial tissue of patients with active inflammatory arthritis (RA and psoriatic arthritis). Additionally, PRL collaborates with pro-inflammatory stimuli to enhance the expression of various cytokines and chemokines in macrophages [23]. Exposure to PRL has also been shown to increase TNF-α release in monocytes from RA patients [24]. In a rodent model of inflammatory arthritis induced by intra-articular injection of cytokines, treatment with high concentrations of PRL or the dopaminergic antagonist haloperidol produced similar results, providing protection against joint destruction by inhibiting chondrocyte apoptosis [25].

Some authors [26, 27] believe that PRL predominantly plays a triggering role in initiating autoimmune diseases, while others [28] believe that it is primarily involved in maintaining inflammatory activity. In this context, it is not possible to overlook the involvement of other hormones such as cortisol and catecholamines. In RA, impairment of hypothalamic-pituitary-adrenal axis activity associated with sympathetic nervous system dysfunction would favor worsening of stress-induced disease activity [29]. Duration and intensity of the stressor stimulus could lead to different actions of hyperprolactinemia. In a situation of hyperprolactinemia induced by chronic stress, PRL would be associated with an immunosuppressive effect, in contrast to the inflammatory effect promoted by hyperprolactinemia induced by acute stress [30].

There is also crosstalk between the susceptibility and severity of RA with sex hormones [31] and other placental hormones [32]. During pregnancy, placental steroid hormones associated with hyperprolactinemia contribute to the effects on RA remission [32, 33], while exacerbation of postpartum disease occurs due to a decline in these hormones and maintenance of hyperprolactinemia [32]. Outside the context of pregnancy, estrogens participate in stimulating a β-cell-mediated immune response but may have anti-inflammatory effects on T cells, macrophages, and other immune cells, in contrast to androgens, which predominantly exhibit immunosuppressive effects [31].

Beyond disease activity, the metabolic impact of PRL has been observed in adipose, hepatic, pancreatic, and brain tissues. Evidence suggests that PRL suppresses lipid storage and the release of adiponectin, IL-6, and potentially leptin [34]. However, an experimental study on rat adipose tissue has demonstrated stimulation of leptin synthesis and secretion [35]. In adipose tissue, the autocrine action of PRL inhibits lipolysis [36]. Another publication suggests the involvement of PRL in brain resistance to leptin, leading to increased food intake [37]. The reduction of dopaminergic tone present in states of hyperprolactinemia also contributes to hyperphagia [36].

In the pancreas, the effects of PRL are most pronounced during pregnancy, involving beta cell proliferation and heightened glucose-stimulated insulin secretion [38, 39]. Conversely, hyperprolactinemic patients exhibit reduced insulin sensitivity and impaired endothelial function [40]. In the liver, the physiological concentration of PRL appears to play a protective role in preventing hepatic steatosis [36].

Previous studies have assessed PRL levels in RA patients compared to control groups without RA [22, 23]. The objective of this exploratory study is to attempt a comparison of PRL concentrations among groups of patients already diagnosed with RA (thus sharing similarities in other disease-specific aspects such as the presence of antibodies, or exposure to RA-specific medication therapy) and who have been followed since the first 12 months of symptom onset, that is, since early RA diagnosis [41, 42]. Thus, the study aimed to examine the presence of changes in serum PRL between patients with moderate to high disease activity (MHDA), comparing them to those with controlled disease activity, defined as remission or low disease activity (RLDA). We assessed whether there was a higher likelihood of clinical outcomes, such as fatigue, morning stiffness, and worse disease activity scores, as well as glycemic dysfunction, across different concentrations of PRL. The identification of PRL as a variable related to clinical and metabolic aspects of RA may contribute to the formulation of more consistent hypotheses regarding the role of this hormone in autoimmune diseases.

## Materials and methods

### Study design and sampling

This is a cross-sectional study that selected female patients diagnosed with RA according to the *American College of Rheumatology/European League Against Rheumatism* - ACR/EULAR 2010 criteria [15] and who participated in the BSB Cohort. The BSB Cohort [41, 42] is an inception cohort, in which all patients initiated follow-up within a year of the onset of symptoms. The individuals in the cohort are closely monitored in accordance with the treatment protocol for RA as outlined by the Brazilian Society of Rheumatology. This monitoring is carried out in alignment with the principles of tight control and treat-to-target strategy. Patients in this cohort demonstrated high adherence to follow-up and therapy, improving disease control and subsequently reducing the need for corticosteroid use [41]. Given the known roles of corticosteroids and estrogens in inflammatory [31, 43] and metabolic responses [19, 44], the characteristics of the selected sample aim to minimize the potential interference of the interactions from other axes with the prolactin axis. This is because the study´s interest is to evaluate the isolated participation of prolactin in the proposed objectives. Pregnant or breastfeeding women, those with liver cirrhosis, and individuals with chronic renal failure and a glomerular filtration rate below 60 ml/min were excluded from participation in the study. Informed consent was obtained from all patients prior to their inclusion in the study. This study was carried out in accordance with the principles of the Declaration of Helsinki and followed the recommendations of resolution 19/2012 of the National Health Council of that country, approved by the research ethics committee of the institution under CAAE 25775819.0.0000.5558, report 3,739.106.

### Patient data collection

Patients were recruited between 2019 and 2020, and the sample size was determined via convenience sampling, considering the accessibility and availability of participants. At the time of recruitment, medical information, clinical examination, and laboratory collection were conducted. Clinical and epidemiological data were collected using standardized questionnaires. The use of medications related to secondary hyperprolactinemia was investigated. The presence of autoantibodies was determined through a review of medical records, with positivity determined in accordance with the specific methodology and kit utilized during the corresponding period. Blood samples were collected in the morning for the purpose of analyzing essential laboratory data. Height (meters) and weight (kilograms) were the measurements taken to compute the body mass index [BMI, BMI = weight (kg)/height (m²)]. The evaluation of patient’s joint conditions was conducted by experienced rheumatologists to calculate composite disease activity indices.

Disease activity was assessed using composite disease activity indices, namely the DAS28-ERS (*Disease Activity Score in 28 joints erythrocyte sedimentation rate*) [45] or CDAI (*clinical disease activity score)*, [46, 47] the latter employed when laboratory assessment of ERS (erythrocyte sedimentation rate) was unavailable. The definition of the group with controlled disease included patients in remission and those with low activity (RLDA group). This approach was based on the goals of the treat-to-target strategy for RA treatment, where the target for disease control is remission, but low disease activity can be considered acceptable, especially in patients with a long disease duration [48]. Patients with composite disease activity indices ranging from moderate to high in the DAS28-ERS or CDAI constituted the group classified as having active inflammation.

Fatigue intensity was gauged using the 0–100 mm visual analogue scale of fatigue (VAS). Scores below 2 mm were deemed clinically insignificant fatigue, while scores ≥ 20 mm were classified as indicative of fatigue. Instances of morning stiffness were recorded based on the self-reported presence of this symptom, irrespective of the disease duration.

### Laboratory tests

Serum PRL was assessed using two distinct methodologies: chemiluminescence immunoassay and electrochemiluminescence immunoassay (ECLIA), both following the instructions provided by the manufacturer and supplier. The chemiluminescence method yielded a lower detection limit of 0.25 ng/ml, while the electrochemiluminescence method had a lower detection limit of 0.047 ng/ml. Given the variability in detection based on the method and the kit employed, the reference mean for prolactin exhibited slight fluctuations across laboratories. Hyperprolactinemia was defined as PRL values exceeding 24 ng/ml (µg/L).

Fasting plasma glucose was determined using an automated UV-hexokinase enzymatic method, following the manufacturer’s guidelines. Considering the methodology, kit specifics, and established diagnostic criteria for glycemic disorders, normal blood glucose values ranged between 70 and 99 mg/dl. Blood glucose values greater than 100 mg/dl were defined as dysglycemia.

The erythrocyte sedimentation rate test, utilized to compute the composite disease activity indices, was conducted using an automated method based on photometric and kinetic principles.

### Statistical analyses

Descriptive and analytical analyses were conducted on the sample. The normality of the distribution for variables characterizing the studied population was assessed using the Shapiro–Wilk test. Depending on the distribution of each variable, parametric measurements (mean and confidence interval [CI]) or, when appropriate, nonparametric measurements (median and interquartile range [IQR]) were used to describe numerical variables. Categorical variables were described in terms of absolute values and the corresponding percentages.

Sample characteristics were compared on the basis of disease activity, distinguishing between RLDA and MHDA groups. The *Student´s t-*test or *Wilcoxon* test were employed for numerical variables, while *chi-square* or *Fisher* tests were utilized for categorical variables. Additionally, laboratory manifestations of the participants were compared in subgroups defined by the presence or absence of hyperprolactinemia with median prolactin values compared through the *Mann–Whitney* test.

To gauge the relationship between blood glucose, fatigue, disease activity, morning stiffness, and categorized PRL values, *chi-square* and *Fisher’s chi-square* tests were applied. Multinomial regression was used to explore associations between PRL categories and dichotomized variables of interest. Within the multinomial regression model, PRL values falling below the first category (< 7.75) were taken as the reference category. In multivariate analysis, all association measures were adjusted for age, and some were further adjusted for variables including time since RA diagnosis, corticosteroid usage, and the presence of positive rheumatoid factor. For the disease activity outcome, the use of oral estrogen was also incorporated.

A significance level of 5% (*p* < 0.05) was employed for all analyses, which were carried out using the statistical software STATA version 14.

## Results

Out of 160 potentially eligible patients, who were regularly followed up at a specialized outpatient clinic, 79 patients were initially selected on the basis of the inclusion and exclusion criteria. Among these, four patients did not provide samples for laboratory evaluation during the recruitment period, and three patients did not undergo the physical examination, resulting in 72 patients who completed the evaluation. No patients included in the study were using antipsychotics, opioids, monoamine oxidase inhibitors, or antiemetics at the time of sample analysis. Twelve patients were identified as using tricyclic antidepressants, and six patients were identified as using serotonin reuptake inhibitors; however, hyperprolactinemia was not detected in any of these patients. The patients in the sample with hypothyroidism had serum TSH values within the normal limits for the method, except for two patients in the controlled disease group (RLDA group) who had slightly elevated TSH levels close to 5µUI/ml. Nevertheless, the two patients with serum TSH levels above the upper limit did not present with hyperprolactinemia. Due to the lack of identification of the mentioned factors in patients with hyperprolactinemia, these conditions did not compromise the comparative analyses between the groups and, therefore, were not included as confounding factors.The participants were predominantly positive for rheumatoid factor with bone radiological changes evident in 47.8%. The clinical and epidemiological characteristics at the time of assessment are described in Table 1.

**Table 1:** Clinical-epidemiological characteristics of women with rheumatoid arthritis (RA) according to inflammatory activity, BSB cohort

Variable	Disease activity					
	Total	Low/Remission	Total	Moderate/High	Total sample	*p*-value*
Age (years), mean (95%CI)	44	56.1 (51.5–60.8)	28	47.5 (42.1–52.9)	52.8 (49.2–56.3)	0.02
Age at diagnosis of RA (years), mean (95%CI)		46.5 (41.1–50.9)		39.1 (33.8–44.3)	43.6 (40.2–47.0)	0.03
Diagnosis of RA during reproductive age, n (%)		22 (50%)		18 (64.3%)	40 (55.6%)	0.34
Time of diagnosis (years), median (IQR)		9.0 (5.5–14.0)		6.5 (2.5–13.0)	9.0 (5.0–14.0)	0.14
Positive rheumatoid factor, n(%)	44	29 (65.9%)	27	23 (85.2%)	52 (72.2%)	0.07
Use of oral estrogen, n(%)	44	9 (20.4%)	28	3 (10.7%)	12 (16.7%)	0.35
Pharmacological therapy, n(%)	42		27			
Monotherapy		16 (38.1%)		10 (37%)	26 (37.7%)	0.31
Combination therapy		25(59.5%)		14 (51.8%)	39 (56.5%)	1.00
Biological agents**		13 (29.6%)		9 (32.1%)	22 (30.6%)	1.00
Glucocorticoids	43	5 (11.6%)	27	13 (48.1%)	18 (25.7%)	0.001
Body mass index (kg/m2), median (IQR)	44	25.3 (21-28.8)	28	26.7 (23.1–30.5)	26.3 (22.3–29.8)	0.28
Obese(%)		22.7%		28.6%	25%	0.60
VAS score for fatigue, median (IQR)	44	0 (0–3)	28	4(0–6)	0(0-4.5)	0.01
0–10		29 (65.9%)		11 (39.3%)	40 (55.6%)	
20–40		9 (20.4%)		5 (17.9%)	14 (19.4%)	
≥50		6 (13.6%)		12 (42.9%)	18 (25%)	
Morning stifness, n(%)	42	7 (16.7%)	27	17 (63%)	24 (34.8%)	< 0.000

*Chi-square test or Fisher’s test were used to test the equality of proportions; Student’s *t*-test to compare means, and Mann-Whitney to test the medians. *95%CI* 95% confidence interval, *IQR* interquartile range

**Infliximab, certolizumab, rituximab, abatacept, etanercept, golimumab, adalimumab, tocilizumab

Only six patients had the CDAI used to assess disease activity, and all these patients were classified in the remission category. Most of the patients were categorized as being in the RLDA group at the time of assessment.

Table 1 provides clinical-epidemiological characteristics of women with RA according to inflammatory activity.

Hyperprolactinemia was displayed in 9.7% of all patients, as shown in Table 2, and the maximum PRL value was 41.5 ng/ml. Considering the variable PRL as a continuous numeric variable, which exhibited a non-normal distribution, the median PRL level was 9.9 ng/ml (IQR 7.7–13.45) in the normoprolactinemic group and 34.4 ng/ml (IQR 25.6–36.7) in the hyperprolactinemia group with *p* < 0.0001. The Table 2 also displays other laboratory characteristics of the studied sample according to disease activity.

**Table 2:** Laboratory characteristics of women with rheumatoid arthritis (RA) according to disease activity, BSB cohort

Variable	Disease activity				Total sample	*P*-value*
	Total	Low/remission	Total	Moderate/high		
Dysglycemia, n (%)	42	14 (33.3%)	28	6 (21.4%)	20 (28.6%)	0.42
Diabetes, n (%)		6 (14.2%)		6 (21.4%)	12 (17.14%)	
Blood glucose (mg/dl), median (IQR)	42	93 (86–101)	28	90 (86.5–98)	91 (86–101)	0.74
Blood glucose < 100		28 (66.7%)		22 (78.6%)	50 (71.4%)	
Blood glucose 100–125		11 (26.2%)		1 (3.6%)	12 (17.1%)	0.02**
Blood glucose ≥ 126		3 (7.1%)		5 (17.9%)	8 (11.4%)	
Serum prolactin (ng/ml), median (IQR)	44	9.9 (6.9–14.5)	28	11.2 (8.6–16.7)	10.3 (7.7–15.2)	0.26
Hyperprolactinemia		4 (9.1%)		3 (10.7%)	7 (9.7%)	1.00

*P-value calculated by Mann–Whitney test to test for median equality and chi-square or Fisher to test equality of proportions. *IQR* interquartile range

**There was no statistical difference between dysglycemia and diabetes comparing the inflammation subgroups (p value of 0.28 and 0.25, respectively)

To investigate the association between hyperprolactinemia and dysglycemia, as well as characteristics such as fatigue and morning stiffness, patients were grouped according to the presence or absence of hyperprolactinemia. No significant results were observed in the comparison between the normal PRL and hyperprolactinemia groups. This information is available in Table 3.

**Table 3:** Characteristics of women with rheumatoid arthritis (RA) according to serum prolactin, BSB cohort

Variable	Serum prolactin				Total sample	*P*-value*
	*n*	Normal prolactin	*N*	Hyperprolactinemia		
Glucose (mg/dl), median (IQR)	63	92 (87–102)	7	86 (74–95)	91 (86–101)	0.04
Glucose < 100		43 (68.2%)		7 (100.0%)	50 (71.4%)	0.35
Glucose 100–125		12 (19.0%)		-	12 (17.1%)	
Glucose ≥ 126		8 (12.7%)		-	8 (11.4%)	
VAS score for fatigue, median (IQR)	65	0 (0–4)	7	2 (0–5)	0 (0–4.5)	0.45
0–1		38 (58.5%)		2 (28.6%)	40 (55.6%)	0.16
2–4		11 (16.9%)		3 (42.9%)	14 (19.4%)	
≥ 5		16 (24.6%)		2 (28.6%)	18 (25%)	
Presence of morning stiffness	62	21 (33.9%)	7	3 (42.9%)	24 (34.8%)	0.69

*IQR* interquartile range

*P-value calculated using the Mann–Whitney test for equality of medians and chi-square or Fisher test for equality of proportions

Subsequently, as can be observed in Table 4, the patients were divided into categories according to serum PRL and compared to a reference category, defined as serum PRL values below 7.5 ng/ml. The analysis of the outcome presence of clinically significant fatigue revealed a higher likelihood of encountering this outcome among patients with intermediate PRL values (prolactin categories 7.76–10.35 and 10.36–15.29) when compared to the reference category. This observation remained consistent even after conducting multivariate analysis. After adjusting for age, the variable normoglycemia showed no statistically significant differences across the various PRL categories.

**Table 4:** Association between categories of prolactin and manifestations in women with rheumatoid arthritis (RA), BSB cohort

Variables	Categories of Prolactin						
	< 7.75	7.76–10.35		10.36–15.29		> 15.30	
	OR	OR	95%CI	OR	95%CI	OR	95%CI
**Univariate analysis**							
Normal blood glucose (< 100)	1.00	1.56	0.39–6.25	1.87	0.44–7.85	13.22	1.40–124.90
Fatigue (VAS ≥ 20 mm)	1.00	5.00	1.06–23.46	7.86	1.65–37.40	5.00	1.06–23.46
Moderate/high disease activity	1.00	2.80	0.66–11.92	2.80	0.66–11.92	2.80	0.66–11.92
Morning stiffness	1.00	0.92	0.23–3.70	1.43	0.35–5.79	0.70	0.17–2.95
**Multivariate analysis** ^**a**^							
Normal blood glucose (< 100)	1.00	1.87	0.40–8.76	1.76	0.34–9.05	5 0.43	0.51–58.28
Fatigue (VAS ≥ 20 mm)	1.00	5.18	1.01–26.38	6.25	1.20–32.51	2.66	0.50–14.24
Disease activity moderate/high	1.00	1.90	0.32–11.49	3.50	0.54–22.81	1.15	0.19–6.89
Morning stiffness	1.00	0.69	0.12–3.66	0.46	0.06–3.44	0.11	0.02–0.80

^a^Disease activity was adjusted for age, use of corticosteroids and estrogens, presence of positive rheumatoid factor, age-adjusted fatigue, corticosteroid use, and age-adjusted dysglycemia; morning stiffness was adjusted for age, time of diagnosis, and corticosteroid use

*Reference category < 7.75 (1st quartile)

## Discussion

### PRL and RA disease activity

The results did not reveal any connection between serum PRL levels and clinical manifestations of RA, such as disease activity.

PRL, an immunomodulatory hormone, exhibited a higher prevalence of hyperprolactinemia in the studied sample compared to that in the general adult population, which is 0.4% [49]. Observations from a previous publication demonstrated that women with RA displayed a higher prevalence of hyperprolactinemia compared to healthy controls [50]. However, as observed in a previous study [51], it was also not possible to establish a link between disease activity and serum PRL levels.

The lack of association between PRL concentration and disease activity further supports the hypothesis proposed in a prior publication [23]. This hypothesis suggests that pituitary PRL might exert a more pronounced influence solely during instances of significant hyperprolactinemia, such as during breastfeeding. Alternatively, it is plausible that its primary role lies in being locally generated at the site of inflammation [23, 52]. This notion finds backing in a study where the expression of the PRL receptor in the joint synovium was increased in inflammatory arthritis [53].

In the current study, PRL values were slightly elevated, in agreement with previous findings that, in general, plasma PRL concentrations in RA are not elevated on average [23, 50]. The plasma concentrations identified may also reflect changes promoted in the regulation of pituitary PRL by inflammatory cytokines found in RA. Experiments in premenopausal women with RA showcase a better response in PRL elevation to the hypoglycemic stimulus induced in the insulin tolerance test (ITT) after TNF inhibition, suggesting that the action of this cytokine attenuates the pituitary PRL response in stressful situations [54].

### PRL and other clinical features of RA

In a cross-sectional investigation, there was no observed association between PRL levels and other variables. However, the low prevalence of certain symptoms, such as morning stiffness, may have compromised the analysis of the association. The majority of patients were in the RLDA group, which explains the low occurrence of this symptom, as it is more easily identifiable as disease activity increases [55]. Therefore, assessing stiffness in the RLDA group, which was predominant in our sample, poses greater challenges [56]. Correlations between distinct PRL concentration categories and morning stiffness were not identified. In a study involving patients with rheumatic polymyalgia, an inflammatory condition, the duration of morning stiffness exhibited a positive correlation with serum PRL levels [57], but there is a lack of literature data correlating this symptom with morning stiffness in RA.

Furthermore, the enrolled patients exhibited lower median fatigue scores than those reported in analogous studies [58, 59]. The literature indicates conflicting findings concerning the association between disease activity and fatigue, suggesting that even controlling disease activity might not fully alleviate fatigue in RA patients [59]. Other factors besides pain, such as mental health, joint deformities, and sleep disturbances, could function as predictive factors for fatigue outcomes [60].

Upon assessment of the correlation of PRL levels and the clinical manifestation of fatigue, a heightened likelihood of experiencing fatigue was observed among patients categorized within the PRL range of 7.75 to 15.29 in comparison to patients whose PRL values were below 7.75. The results obtained may have been affected by the small number of patients with clinically significant fatigue in the sample. There is a shortage of literature establishing a connection between fatigue exhibited in cases of RA and serum PRL levels. However, one study identified decreased activity of the dopaminergic system in the basal ganglia of patients with chronic fatigue syndrome. This syndrome exhibits similarities with RA regarding inflammation, as elevated markers of immune activation were observed in patients with chronic fatigue syndrome [61]. It is important to emphasize that hypoactivity of the dopaminergic system was identified in specific regions of the basal ganglia in this population, and a comparable impact on the hypothalamic nuclei responsible for PRL control could not be identified.

Other aspects need to be considered when assessing the symptoms of fatigue. Additional investigations, in diseases with characteristics similar to RA, such as the previously mentioned chronic fatigue syndrome, have established a correlation between the presence of fatigue syndrome and a disruption in the diurnal fluctuations of cortisol, rather than overall cortisol production [62, 63]. This aligns with the observation of symptom improvement in patients with adrenal insufficiency when using extended-release hydrocortisone, a presentation that provides a more physiological cortisol release [64]. Within the BSB cohort, a significant number of patients were observed to be using corticosteroids in the higher PRL level ranges. It is important to note that a previous publication involving RA patients identified adrenal insufficiency in 48% of individuals using prednisolone at dosage of 5 mg/day for a least 6 months [65]. Thus, the possibility of a significant proportion of adrenal insufficiency cases within the group of women using corticosteroids cannot be disregarded. The presence of hypothalamic–pituitary–adrenal (HPA) axis dysfunction among the subset of patients exhibiting elevated PRL values could potentially contribute to more pronounced fatigue scores within this subgroup. In the multivariate analysis, after adjustment for the variable corticosteroid use, the association between increased fatigue and PRL levels persisted only within the intermediate PRL categories, relative to the reference category, affecting the causality hypothesis due to the loss of biological gradient. It is worth considering, however, that different PRL levels in different pathological conditions may yield divergent results. In an experiment that observed the expression of different cytokines based on varying PRL concentrations, the results were attributed to different modes of PRL receptor binding. At physiological levels, PRL would promote receptor dimerization, leading to activation, whereas at elevated PRL, each receptor would be activated, impairing post-receptor signaling [66].

### PRL and metabolic changes in RA

The percentage of obesity within the studied sample did not diverge from the percentage observed in a cohort of Brazilian patients with RA [67]. As previously mentioned, in the context of elevated PRL, which was observed in a very small portion of the sample, various mechanisms act to favor increased appetite, inhibit lipolysis, and consequently lead to obesity [36].

Analysis of serum glucose levels among the patients within the sample revealed that most of them had blood glucose levels below 100 mg/dl. In the association of PRL with glycemic outcomes, the number of publications [68–70] describing various metabolic results within the range considered physiological for PRL levels has been increasing. Markedly suppressed concentrations of serum PRL appear to increase the risk of diabetes [70]. Other findings supporting the association of metabolic dysfunction with low prolactin levels include the observation that obese patients have damped prolactin secretion [71]. Remarkably, higher quartiles of PRL correlate with enhanced insulin sensitivity and lower plasma glucose values [72, 73]. A similar pattern has been observed among women dealing with infertility and polycystic ovary syndrome; in this context, lower serum PRL concentrations have been identified as a metabolic risk factor, [74] concomitant with insulin resistance and β-cell dysfunction [75].

When the evaluated sample was stratified into categories, no difference was observed in the likelihood of finding serum glucose below 100 mg/dl when a specific PRL category was compared to the category corresponding to the lowest quartile of PRL. Considering that the lowest quartile corresponded to PRL levels below 7.75 ng/ml, even when compared to a metabolically unfavorable value, no favorable results were observed for higher PRL levels. On the other hand, mildly elevated PRL levels between 25 and 100 µg/L, in the absence of a pathological cause, would be considered beneficial for metabolic homeostasis, and this range of values is referred to as “homeostatic functionally increased transient prolactinemia” (HomeoFIT-PRL) [68, 69]. In this study, patients with hyperprolactinemia had PRL values within the HomeoFIT-PRL range, so the observation that all hyperprolactinemic patients maintained glucose levels below 100 mg/dl is in line with expectations.

However, higher concentrations of PRL, such as those observed during pregnancy, have been associated with poorer plasma glucose values, and a significant impairment of glucose tolerance is particularly noted in the third trimester– the peak period of PRL levels [76]. Nevertheless, this observation contradicts the finding of a recently published study [77] that revealed that median PRL levels in the third trimester were inversely associated with the risk of gestational diabetes. As mentioned earlier, it is believed that different PRL concentrations may interact differently with the receptor, compromising the expected effects [66].

In the context of pathological hyperprolactinemia, as found in patients with PRL-secreting pituitary adenoma, it was possible to identify that the use of dopaminergic agonists contributed favorably to glycemic homeostasis, improving insulin resistance, independent of the percentage reduction in PRL concentration [78, 79]. This underscores the role of dopamine´s direct action on receptors presents in extra-pituitary tissues directly participating in metabolic homeostasis [36].

## Conclusions

Several limitations should be acknowledged within the scope of this study. As it was an exploratory cross-sectional study, establishing causal relationships between the described observations was not feasible. Furthermore, the sample size could have impacted the results. To ensure a larger and more diverse sample in terms of inflammatory activity, future studies can include additional centers. The selected cohort in this study consisted of patients with controlled disease activity and a lower incidence of fatigue and morning stiffness, which may have influenced the results. Despite these limitations, the study has provided some insights that may contribute to discussions about the interplay between the neuroendocrine and immune systems in the context of RA. No association was identified between PRL levels and worse scores for disease activity in RA. Regarding the influence of various PRL levels on glycemic homeostasis, the study did not yield robust evidence to support previously established observations on the subject. Efforts should be made to reproduce the results in other cohorts and using different methodologies. Regarding fatigue, it was not possible to establish conclusive evidence linking this symptom to serum PRL. However, the symptom of fatigue was assessed only by the VAS, and other existing specific questionnnaries were not used. Nevertheless, the observation of a higher likelihood of finding this symptom in RA patients with serum PRL levels between 7.75 and 15.29 when compared to those with PRL levels below this range is noteworthy. Since fatigue remains a poorly understood symptom, these data may serve as a catalyst for sparking further discussions.

Future longitudinal studies that observe individual changes in serum PRL and its correlation with clinical and metabolic manifestations over time could contribute to a better understanding of the role of PRL in RA. Expanding knowledge on novel mechanisms involved in immune-mediated disease is of paramount importance, emphasizing the need for a more comprehensive understanding of central and peripheral hormonal effects on autoimmune disease manifestations. These effects have the potential to influence the progression of RA diseases and may even unveil novel therapeutic opportunities.

## Electronic supplementary material

Below is the link to the electronic supplementary material.

Supplementary Material 1

## Data Availability

The datasets generated and/or analyzed during the current study are available in the UNB repository, http://repositorio2.unb.br/jspui/handle/10482/43037.

## Abbreviations

PRL: Prolactin
RA: Rheumatoid arthritis
RLDA: Remission and low disease activity
MHDA: Moderate to high disease activity
