# Supplementary material for: Exploring the association of serum prolactin with serum glucose levels and clinical findings in a cohort of patients with early rheumatoid arthritis
Source: Adv Rheumatol. 2024 Aug 7;64:56. doi: 10.1186/s42358-024-00394-8 (PMC13488692; doi:10.1186/s42358-024-00394-8)
Supplement: Supplementary file 1 [file 42358_2024_394_MOESM1_ESM.doc]

STROBE Statement—Checklist of items that should be included in reports of ***cross-sectional studies***

|  | Item No | Recommendation |
| --- | --- | --- |
| **Title and abstract** | 1 | (*a*) Indicate the study’s design with a commonly used term in the title or the abstract: Exploring the association of serum prolactin with serum glucose levels and clinical findings in a cohort of patients with early Rheumatoid Arthritis |
|  |  | (*b*) Provide in the abstract an informative and balanced summary of what was done and what was found: **Methods** This exploratory, cross-sectional, observational study focused on women diagnosed with rheumatoid arthritis. The research involved assessing prolactin and blood glucose concentrations, alongside specific clinical traits such as disease-related inflammation, morning stiffness, and fatigue intensity. The presence of changes in serum PRL was initially compared among the groups based on disease activity intensity. Using a multinomial regression analysis, the study analysed the impact of predetermined clinical and metabolic factors on various categories of prolactin concentration.  **Results** Out of the 72 participants included in the study, hyperprolactinemia was detected in 9.1% of the sample. No differences in serum PRL were identified among the evaluated groups based on disease activity. Following multivariate analysis, no statistically significant differences were identified for the outcomes of inflammatory activity and morning stiffness within each PRL category when compared to the reference category for PRL. There was no increased likelihood of encountering blood glucose levels below 100 mg/dl among individuals with higher prolactin concentrations compared to those in the lowest prolactin category (OR 5.43, 95% CI 0.51–58.28). The presence of clinically significant fatigue revealed a higher likelihood of encountering this outcome among patients with intermediate PRL values (prolactin categories 7.76–10.35 with OR 5.18, 95% CI 1.01–26.38 and 10.36–15.29 with OR 6.25, 95% CI 1.2–32.51) when compared to the reference category. |
| Introduction | | |
| Background/rationale | 2 | Explain the scientific background and rationale for the investigation being reported: In the context of rheumatoid arthritis and its systemic inflammatory implications, there is an increasing interest in investigating the role of prolactin in the clinical and metabolic aspects of the disease. PRL has been associated with numerous biological functions over the years, particularly in the context of metabolic and immunological activities. In addition to pituitary PRL, other sources of this hormone have been identified, including cells of the immune system. Furthermore, distinct actions of PRL can be observed based on the concentration of this hormone, for example, increased metabolic risk in individuals with supressed serum prolactin levels. This study aimed to explore the potential links between serum prolactin levels, serum glucose levels, and the clinical manifestations of arthritis |
| Objectives | 3 | State specific objectives, including any prespecified hypotheses: The objective of this exploratory study is to attempt a comparison of PRL concentrations among groups of patients already diagnosed with RA (thus sharing similarities in other disease-specific aspects such as the presence of antibodies, or exposure to RA-specific medication therapy). Thus, the study aimed to examine the presence of changes in serum PRL between patients with moderate to high disease activity (MHDA), comparing them to those with controlled disease activity, defined as remission or low disease activity (RLDA). We assessed whether there was a higher likelihood of clinical outcomes, such as fatigue, morning stiffness, and worse disease activity scores, as well as glycaemic dysfunction, across different concentrations of PRL |
| Methods | | |
| Study design | 4 | Present key elements of study design early in the paper: At the outset of the article, the aim was to underscore the role of prolactin in the immunological and metabolic context, to justify the choice of this hormone as the study subject in rheumatoid arthritis. In the introduction, the role of systemic inflammation in rheumatoid arthritis patient´ metabolic risk was also addressed. Previous publications associating prolactin with inflammatory activity, as well as with metabolic risk, were cited. All existing publications compare rheumatoid arthritis patients with healthy controls or controls with non-inflammatory disease, contrasting in the study´s proposal, which includes only individuals with rheumatoid arthritis across different spectra of inflammation |
| Setting | 5 | Describe the setting, locations, and relevant dates, including periods of recruitment, exposure, follow-up, and data collection: This is a cross-sectional study that selected female patients diagnosed with RA according to the *American College of Rheumatology/European League Against Rheumatism* - ACR/EULAR 2010 criteria and who participated in the BSB Cohort. The BSB Cohort is an inception cohort, in which all patients initiated follow-up within a year of the onset of symptoms. The individuals in the cohort are closely monitored in accordance with the treatment protocol for RA as outlined by the Brazilian Society of Rheumatology. This monitoring is carried out in alignment with the principles of tight control and treat-to-target strategy.Patients were recruited between 2019 and 2020 |
| Participants | 6 | (*a*) Give the eligibility criteria, and the sources and methods of selection of participants: This study selected female patients diagnosed with RA according to the *American College of Rheumatology/European League Against Rheumatism* - ACR/EULAR 2010 criteria and who participated in the BSB Cohort. Pregnant or breastfeeding women, those with liver cirrhosis, and individuals with chronic renal failure and a glomerular filtration rate below 60 ml/min were excluded from participation in the study. Informed consent was obtained from all patients prior to their inclusion in the study.The sample size was determined via convenience sampling, considering the accessibility and availability of participants |
| Variables | 7 | Clearly define all outcomes, exposures, predictors, potential confounders, and effect modifiers. Give diagnostic criteria, if applicable: The definition of rheumatoid arthritis complies with the criteria of *American College of Rheumatology/European League Against Rheumatism* - ACR/EULAR 2010.  Disease activity was assessed using composite disease activity indices, namely the DAS28-ERS (*Disease Activity Score in 28 joints erythrocyte sedimentation rate*) or CDAI (*clinical disease activity* *score)*, the latter employed when laboratory assessment of ERS (erythrocyte sedimentation rate) was unavailable. The definition of the group with controlled disease included patients in remission and those with low activity (RLDA group). This approach was based on the goals of the treat-to-target strategy for RA treatment, where the target for disease control is remission, but low disease activity can be considered acceptable, especially in patients with a long disease duration ^(48)^. Patients with composite disease activity indices ranging from moderate to high in the DAS28-ERS or CDAI constituted the group classified as having active inflammation.  Fatigue intensity was gauged using the 0–10 mm visual analogue scale of fatigue (VAS). Scores below 2 mm were deemed clinically insignificant fatigue, while scores ≥2 mm were classified as indicative of fatigue. Instances of morning stiffness were recorded based on the self-reported presence of this symptom, irrespective of the disease duration.  Definition of BMI = weight (kg)/height (m²)  Potential confounding factors assessed age, use of corticosteroids and estrogen, presence of positive rheumatoid factor (The presence of autoantibodies was determined through a review of medical records, with positivity determined in accordance with the specific methodology and kit utilized during the corresponding period), and time of diagnosis  Serum PRL was assessed using two distinct methodologies: chemiluminescence immunoassay and electrochemiluminescence immunoassay (ECLIA), both following the instructions provided by the manufacturer and supplier. The chemiluminescence method yielded a lower detection limit of 0.25 ng/ml, while the electrochemiluminescence method had a lower detection limit of 0.047 ng/ml. Given the variability in detection based on the method and the kit employed, the reference mean for prolactin exhibited slight fluctuations across laboratories. Hyperprolactinemia was defined as PRL values exceeding 24 ng/ml (µg/L).  Fasting plasma glucose was determined using an automated UV-hexokinase enzymatic method, following the manufacturer's guidelines. Considering the methodology, kit specifics, and established diagnostic criteria for glycaemic disorders, normal blood glucose values ranged between 70 and 99 mg/dl. Blood glucose values greater than 100 mg/dl were defined as dysglycemia |
| Data sources/ measurement | 8* | For each variable of interest, give sources of data and details of methods of assessment (measurement). Describe comparability of assessment methods if there is more than one group: At the time of recruitment, medical information, clinical examination, and laboratory collection were conducted. Clinical and epidemiological data were collected using standardized questionnaires. The presence of autoantibodies was determined through a review of medical records, with positivity determined in accordance with the specific methodology and kit utilized during the corresponding period. Blood samples were collected in the morning for the purpose of analysing essential laboratory data. Height (meters) and weight (kilograms) were the measurements taken to compute the body mass index. The evaluation of patient’s joint conditions was conducted by experienced rheumatologists to calculate composite disease activity indices. Disease activity was assessed using composite disease activity indices, namely the DAS28-ERS (*Disease Activity Score in 28 joints erythrocyte sedimentation rate*) ^(45)^ or CDAI (*clinical disease activity* *score)*, ^(46,47)^ the latter employed when laboratory assessment of ERS (erythrocyte sedimentation rate) was unavailable. Fatigue intensity was gauged using the 0–10 mm visual analogue scale of fatigue (VAS). Instances of morning stiffness were recorded based on the self-reported presence of this symptom, irrespective of the disease duration. Serum PRL was assessed using two distinct methodologies: chemiluminescence immunoassay and electrochemiluminescence immunoassay (ECLIA), both following the instructions provided by the manufacturer and supplier. The chemiluminescence method yielded a lower detection limit of 0.25 ng/ml, while the electrochemiluminescence method had a lower detection limit of 0.047 ng/ml. Given the variability in detection based on the method and the kit employed, the reference mean for prolactin exhibited slight fluctuations across laboratories. Hyperprolactinemia was defined as PRL values exceeding 24 ng/ml (µg/L).  Fasting plasma glucose was determined using an automated UV-hexokinase enzymatic method, following the manufacturer's guidelines. Considering the methodology, kit specifics, and established diagnostic criteria for glycaemic disorders, normal blood glucose values ranged between 70 and 99 mg/dl. Blood glucose values greater than 100 mg/dl were defined as dysglycemia.  The erythrocyte sedimentation rate test, utilized to compute the composite disease activity indices, was conducted using an automated method based on photometric and kinetic principles |
| Bias | 9 | Describe any efforts to address potential sources of bias: Given the known roles of corticosteroids and estrogen in inflammation, the sample restriction to only female patients occurred in an attempt to avoid bias related to the presence or absence of estrogen exposure. The choice of the BSB cohort aimed to minimize the interference of corticosteroid use, as patients in this cohort typically demonstrate high adherence to follow-up and therapy, generally experiencing good control of inflammatory activity and thus reducing the need for corticosteroid use.  The presence of uncompensated hypothyroidism and the use of major drugs that can induce hyperprolactinemia, such as tricyclic antidepressants, serotonin reuptake inhibitors, antipsychotics, opioids, monoamine oxidase inhibitors and antiemetics, were investigated in the evaluated sample. No patients included in the study were using antipsychotics, opioids, monoamine oxidase inhibitors, or antiemetics at the time of sample analysis. Twelve patients were identified as using tricyclic antidepressants, and six patients were identified as using serotonin reuptake inhibitors; however, hyperprolactinemia was not detected in any of these patients. The patients in the sample with hypothyroidism had serum TSH values within the normal limits for the method, except for two patients in the controlled disease group (RLDA group) who had slightly elevated TSH levels above 5µUI/ml. Nevertheless, the two patients with serum TSH levels above the upper limit did not present with hyperprolactinemia. Due to the lack of identification of the mentioned factors in patients with hyperprolactinemia, these conditions did not compromise the comparative analyses between the groups and, therefore, were not included as confounding factors.Furthermore, during statistical analysis, in the multivariate analysis, all association measures were adjusted for age, and some were further adjusted for variables including time since RA diagnosis, corticosteroid usage, and the presence of positive rheumatoid factor. For the disease activity outcome, the use of oral estrogen was also incorporated |
| Study size | 10 | Explain how the study size was arrived at: Among the eligible study participants the sample size was determined through convenience sampling, considering the accessibility and availability of participants. The sample size for our study was determined to ensure 80% statistical power, with an anticipated effect size of 0.8, a significance level (alpha) of 0.05, and a standard deviation of 2. This requires approximately 26 participants per group. This sample size calculation is based on a two-tailed test |
| Quantitative variables | 11 | Explain how quantitative variables were handled in the analyses. If applicable, describe which groupings were chosen and why. The quantitative variables from the composite disease activity indices were used for classification of the inflammatory groups. The definition of the group with controlled disease included patients in remission and those with low activity (RLDA group). This approach was based on the goals of the treat-to-target strategy for RA treatment, where the target for disease control is remission, but low disease activity can be considered acceptable, especially in patients with a long disease duration ^(48)^. Patients with composite disease activity indices ranging from moderate to high in the DAS28-ERS or CDAI constituted the group classified as having active inflammation  Fatigue intensity was gauged using the 0–100 mm visual analogue scale of fatigue (VAS). Scores below 2 mm were deemed clinically insignificant fatigue, while scores ≥20 mm were classified as indicative of fatigue  Hyperprolactinemia was defined as PRL values exceeding 24 ng/ml (µg/L)  Blood glucose values greater than 100 mg/dl were defined as dysglycemia.  The normality of the distribution for variables characterizing the studied population was assessed using the Shapiro–Wilk test. Depending on the distribution of each variable, parametric measurements (mean and confidence interval [CI]) or, when appropriate, nonparametric measurements (median and interquartile range [IQR]) were used to describe numerical variables  Sample characteristics were compared on the basis of disease activity, distinguishing between RLDA and MHDA groups. The *Student´s t-*test or *Wilcoxon* test were employed for numerical variables |
| Statistical methods | 12 | (*a*) Describe all statistical methods, including those used to control for confounding: Descriptive and analytical analyses were conducted on the sample. The normality of the distribution for variables characterizing the studied population was assessed using the Shapiro–Wilk test. Depending on the distribution of each variable, parametric measurements (mean and confidence interval [CI]) or, when appropriate, nonparametric measurements (median and interquartile range [IQR]) were used to describe numerical variables. Categorical variables were described in terms of absolute values and the corresponding percentages.  Sample characteristics were compared on the basis of disease activity, distinguishing between RLDA and MHDA groups. The *Student´s t-*test or *Wilcoxon* test were employed for numerical variables, while *chi-square* or *Fisher* tests were utilized for categorical variables. Additionally, laboratory manifestations of the participants were compared in subgroups defined by the presence or absence of hyperprolactinemia with median prolactin values compared through the *Mann–Whitney* test.  To gauge the relationship between blood glucose, fatigue, disease activity, morning stiffness, and categorized PRL values, *chi-square* and *Fisher’s chi-square* tests were applied. Multinomial regression was used to explore associations between PRL categories and dichotomized variables of interest. Within the multinomial regression model, PRL values falling below the first category (<7.75) were taken as the reference category. In multivariate analysis, all association measures were adjusted for age, and some were further adjusted for variables including time since RA diagnosis, corticosteroid usage, and the presence of positive rheumatoid factor. For the disease activity outcome, the use of oral estrogen was also incorporated.  A significance level of 5% (p < 0.05) was employed for all analyses, which were carried out using the statistical software STATA version 14.: |
|  |  | (*b*) Describe any methods used to examine subgroups and interactions: Sample characteristics were compared on the basis of disease activity, distinguishing between RLDA and MHDA groups. The *Student´s t-*test or *Wilcoxon* test were employed for numerical variables, while *chi-square* or *Fisher* tests were utilized for categorical variables. Additionally, laboratory manifestations of the participants were compared in subgroups defined by the presence or absence of hyperprolactinemia with median prolactin values compared through the *Mann–Whitney* test |
|  |  | (*c*) Explain how missing data were addressed: Patients who lacked data such as serum prolactin or composite disease activity index were excluded from the study, meaning their data were not included in any analysis conducted. For general clinical-epidemiological characteristics and plasma glucose values, data analysis considered the percentage relative to the total available data. |
|  |  | (*d*) If applicable, describe analytical methods taking account of sampling strategy: Subgroup analyses were conducted based on inflammatory activity, and in a secondary analysis based on the presence of hyperprolactinemia. Subsequently, analysis was performed on subgroups according to prolactin categories |
|  |  | (*e*) Describe any sensitivity analyses: In this study, a multinomial regression statistical model was used to explore associations between PRL categories and dichotomized variables of interest, and in the multivariate analysis, all associations measures were adjusted for potential confounding variables |
| Results | | |
| Participants | 13* | (a) Report numbers of individuals at each stage of study—eg numbers potentially eligible, examined for eligibility, confirmed eligible, included in the study, completing follow-up, and analysed: Out of 160 potentially eligible patients, who were regularly followed up at a specialized outpatient clinic, 79 patients were initially selected on the basis of the inclusion and exclusion criteria. Among these, four patients did not provide samples for laboratory evaluation during the recruitment period, and three patients did not undergo the physical examination, resulting in 72 patients who completed the evaluation |
|  |  | (b) Give reasons for non-participation at each stage: Among 79 patients, four patients did not provide samples for laboratory evaluation during the recruitment period, and three patients did not undergo the physical examination, resulting in 72 patients who completed the evaluation |
|  |  | (c) Consider use of a flow diagram: Due to the simplicity of the selection model, it was chosen to describe it in the body of the text, foregoing the flowchart |
| Descriptive data | 14* | (a) Give characteristics of study participants (eg demographic, clinical, social) and information on exposures and potential confounders: The participants had an average age of 52.8 years (95%CI 49.2–56.3) and were predominantly positive for rheumatoid factor (72.2%), with bone radiological changes evident in 47.8%. The median total disease duration for the included patients was nine years (IQR 5.5–14.0) and 55.6% were diagnosed at reproductive age. The majority did not experience morning stiffness (65.8%) or clinically significant fatigue (55.6%) at the time of assessment. Of the total sample, 25% had obesity  Only six patients had the CDAI used to assess disease activity, and all these patients were classified in the remission category. Among the patients selected from the BSB cohort, 44 (61.1%) were categorized as being in the RLDA group at the time of assessment, while 28 (38.9%) were in the MHDA group. The mean age of the patients in the RLDA subgroup was 56.1 (95%CI 51.5–60.8), compared to 47.5 (95%CI 42.1–52.9) for patients in the MHDA subgroup, with a p-value of 0.02. Moreover, patients in the MHDA group were diagnosed at a younger age, with a mean age of 39.1 (95% CI 33.8–44.3), compared to 46.5 (95%CI 41.1–50.9) for patients in the RLDA subgroup (p = 0.03).  A majority of the patients (56.5%) received combination therapy involving two or more medications for treating RA, with approximately 30% of the total patient sample using biological agents. Comparatively, patients with MHDA received glucocorticoids more frequently than those in the RLDA subgroup (48.1% vs 11.6%, p = 0.00). In the selected sample, the disease activity groups showed no significant differences in the percentage of menopausal patients or in the use of exogenous estrogen, whether as hormone replacement therapy (HRT) or as contraception.  Around 44.4% exhibited clinically significant fatigue, and the median fatigue score for the MHDA group was approximately 4, compared to 0 in the RLDA subgroup, yielding a p-value of 0.01. Self-reported morning stiffness was observed in 34.8% of patients. Remarkably, the MHDA subgroup exhibited a higher percentage of morning stiffness, accounting for 63% of occurrences, while the RLDA subgroup had only 16.7% reporting this symptom (p < 0.01). Other assessed aspects, such as time since diagnosis, the presence of positive rheumatoid factor, or obesity, did not differ among the groups. |
|  |  | (b) Indicate number of participants with missing data for each variable of interest: As stated in the tables, variables with missing data were: 1) positive rheumatoid factor ( 1 patient from the MHDA group did not have this test result), 2) serum glucose (2 patients from RLDA group were unable to collect the test at the time of assessment), 3) presence of morning stiffness (2 patients from the RLDA group and 1 patient from the MHDA group were unable to provide objective data for this question at the time of assessment),4) pharmacological therapy (2 patients from the RLDA group and 1 patient from the MHDA group were unable to accurately report all medications used at the time of assessment,) 5) corticosteroid use ( 1 patient from RLDA group and 1 patient from MHDA group were unable to accurately answer if they were using this class of drugs at the time of assessment) |
| Outcome data | 15* | Report numbers of outcome events or summary measures: Out of the 72 participants included in the study, hyperprolactinemia was detected in 9.1% of the sample, Among the patients selected from the BSB cohort, 44 (61.1%) were categorized as being in the RLDA group at the time of assessment, while 28 (38.9%) were in the MHDA group. Around 44.4% exhibited clinically significant fatigue and self-reported morning stiffness was observed in 34.8% of patients. |
| Main results | 16 | (a) Give unadjusted estimates and, if applicable, confounder-adjusted estimates and their precision (eg, 95% confidence interval). Make clear which confounders were adjusted for and why they were included: There was no statistically significant difference between the inflammatory activity subgroups regarding characteristics such as time since diagnosis, diagnosis during reproductive age, estrogen use, pharmacological therapy, BMI, presence of dysglycemia, and presence of hyperprolactinemia. The MHDA group presented a significantly higher percentage of positive rheumatoid factor (p-value 0.07, 95%CI, corticosteroid usage (p-value 0.001, 95%CI), higher fatigue scores (p-value 0.01, 95%CI) and presence of morning stiffness (p-value <0.01, 95%CI). The MDHA group had a significantly lower age mean (p-value 0,02, 95%CI) and younger age at diagnosis (p-value 0,03, 95%CI). The hyperprolactinemia group showed a lower median of serum glucose (p-value 0.04). The patients were divided into categories according to serum PRL and compared to a reference category, defined as serum PRL values below 7.5 ng/ml. No statistically significant differences were identified for the outcomes of inflammatory activity and morning stiffness within each PRL category when compared to the reference category. The analysis of the outcome presence of clinically significant fatigue revealed a higher likelihood of encountering this outcome among patients with intermediate PRL values (prolactin categories 7.76–10.35 and 10.36–15.29) when compared to the reference category. This observation remained consistent even after conducting multivariate analysis. After adjusting for age, the variable normoglycemia showed no statistically significant differences across the various PRL categories |
|  |  | (*b*) Report category boundaries when continuous variables were categorized: Within the multinomial regression model, PRL values falling below the first category (<7.75) were taken as the reference category. The second category included patients with prolactin values between 7.76 and 10.35, the third category included values between 10.36 and 15.29, and the last category included values greater than 15.30 |
|  |  | (*c*) If relevant, consider translating estimates of relative risk into absolute risk for a meaningful time period: not relevant |
| Other analyses | 17 | Report other analyses done—eg analyses of subgroups and interactions, and sensitivity analyses: To gauge the relationship between blood glucose, fatigue, disease activity, morning stiffness, and categorized PRL values, *chi-square* and *Fisher’s chi-square* tests were applied. Multinomial regression was used to explore associations between PRL categories and dichotomized variables of interest. Within the multinomial regression model, PRL values falling below the first category (<7.75) were taken as the reference category. In multivariate analysis, all association measures were adjusted for age, and some were further adjusted for variables including time since RA diagnosis, corticosteroid usage, and the presence of positive rheumatoid factor. For the disease activity outcome, the use of oral estrogen was also incorporated. No statistically significant differences were identified for the outcomes of inflammatory activity and morning stiffness within each PRL category when compared to the reference category. The analysis of the outcome presence of clinically significant fatigue revealed a higher likelihood of encountering this outcome among patients with intermediate PRL values (prolactin categories 7.76–10.35 and 10.36–15.29) when compared to the reference category. This observation remained consistent even after conducting multivariate analysis. After adjusting for age, the variable normoglycemia showed no statistically significant differences across the various PRL categories |
| Discussion | | |
| Key results | 18 | Summarise key results with reference to study objectives: Hyperprolactinemia was detected in 9.1% of the sample. No differences in serum PRL were identified among the evaluated groups based on disease activity. Following multivariate analysis, no statistically significant differences were identified for the outcomes of inflammatory activity and morning stiffness within each PRL category when compared to the reference category for PRL. There was no increased likelihood of encountering blood glucose levels below 100 mg/dl among individuals with higher prolactin concentrations compared to those in the lowest prolactin category (OR 5.43, 95% CI 0.51–58.28). The presence of clinically significant fatigue revealed a higher likelihood of encountering this outcome among patients with intermediate PRL values (prolactin categories 7.76–10.35 with OR 5.18, 95% CI 1.01–26.38 and 10.36–15.29 with OR 6.25, 95% CI 1.2–32.51) when compared to the reference category |
| Limitations | 19 | Discuss limitations of the study, taking into account sources of potential bias or imprecision. Discuss both direction and magnitude of any potential bias: Several limitations should be acknowledged within the scope of this study. As it was an exploratory cross-sectional study, establishing causal relationships between the described observations was not feasible. Furthermore, the sample size could have impacted the results, so when the sample was categorized, the resulting groups had even smaller sample sizes |
| Interpretation | 20 | Give a cautious overall interpretation of results considering objectives, limitations, multiplicity of analyses, results from similar studies, and other relevant evidence: The study has provided some insights that may contribute to discussions about the interplay between the neuroendocrine and immune systems in the context of RA. No association was identified between PRL levels and worse scores for disease activity in RA. Regarding the influence of various PRL levels on glycemic homeostasis, the study did not yield robust evidence to support previously established observations on the subject. Regarding fatigue, it was not possible to establish conclusive evidence linking this symptom to serum PRL. However, the observation of a higher likelihood of finding this symptom in RA patients with serum PRL levels between 7.75 and 15.29 when compared to those with PRL levels below this range is noteworthy. Since fatigue remains a poorly understood symptom, these data may serve as a catalyst for sparking further discussions |
| Generalisability | 21 | Discuss the generalisability (external validity) of the study results. The findings of this study may contribute as a basis for more robust research aiming to establish an association between prolactin and fatigue. The observed results should not be extrapolated to the population with rheumatoid arthritis in general, Studies with more rigorous designs and higher levels of evidence can be conducted based on the initial observations presented here. |
| Other information | | |
| Funding | 22 | Give the source of funding and the role of the funders for the present study and, if applicable, for the original study on which the present article is based: Funding for performing laboratory experiments was provided by the Research Support Center at Sabin Laboratory Brasilia, DF. However, the laboratory had no involvement in the study design, data interpretation, manuscript writing or publication decision |

*Give information separately for exposed and unexposed groups.

**Note:** An Explanation and Elaboration article discusses each checklist item and gives methodological background and published examples of transparent reporting. The STROBE checklist is best used in conjunction with this article (freely available on the Web sites of PLoS Medicine at http://www.plosmedicine.org/, Annals of Internal Medicine at http://www.annals.org/, and Epidemiology at http://www.epidem.com/). Information on the STROBE Initiative is available at [www.strobe-statement.org](http://www.strobe-statement.org/).

The article explores the interrelations between immune-mediated diseases such as rheumatoid arthritis and the endocrine system. The extra-pituitary actions of the hormone prolactin on the immune and metabolic systems were highlighted in this context, emphasizing the potential role of this hormone as a biomarker for clinical and laboratory manifestations of rheumatoid arthritis. The exploratory study did not confirm the finding, which has been described in other publications, where suppressed concentrations of prolactin would act as a metabolic risk factor. In the study, an increase in serum prolactin levels also did not demonstrate an association with inflammatory disease activity, but suggests that in certain ranges of serum prolactin, there may be some association with the clinical manifestations of fatigue. Ultimately, the study is original and may contribute to new research approaches regarding the involvement of prolactin in rheumatoid arthritis.

The manuscript was approved by all authors for submission, and the manuscript has not been published or is not under consideration for publication elsewhere.
